# A Deep Network Model on Dynamic Functional Connectivity With Applications to Gender Classification and Intelligence Prediction

**DOI:** 10.3389/fnins.2020.00881

**Published:** 2020-08-18

**Authors:** Liangwei Fan, Jianpo Su, Jian Qin, Dewen Hu, Hui Shen

**Affiliations:** College of Intelligence Science and Technology, National University of Defense Technology, Changsha, China

**Keywords:** dynamic functional connectivity (dFC), deep learning, gender classification, intelligence prediction, resting-state functional magnetic resonance imaging

## Abstract

Increasing evidence has suggested that the dynamic properties of functional brain networks are related to individual behaviors and cognition traits. However, current fMRI-based approaches mostly focus on statistical characteristics of the windowed correlation time course, potentially overlooking subtle time-varying patterns in dynamic functional connectivity (dFC). Here, we proposed the use of an end-to-end deep learning model that combines the convolutional neural network (CNN) and long short-term memory (LSTM) network to capture temporal and spatial features of functional connectivity sequences simultaneously. The results on a large cohort (Human Connectome Project, *n* = 1,050) demonstrated that our model could achieve a high classification accuracy of about 93% in a gender classification task and prediction accuracies of 0.31 and 0.49 (Pearson’s correlation coefficient) in fluid and crystallized intelligence prediction tasks, significantly outperforming previously reported models. Furthermore, we demonstrated that our model could effectively learn spatiotemporal dynamics underlying dFC with high statistical significance based on the null hypothesis estimated using surrogate data. Overall, this study suggests the advantages of a deep learning model in making full use of dynamic information in resting-state functional connectivity, and highlights the potential of time-varying connectivity patterns in improving the prediction of individualized characterization of demographics and cognition traits.

## Introduction

Functional organization principles in the human brain derived from resting-state functional MRI (rs-fMRI) data have been found to improve our understanding of individual cognition and behavioral differences greatly. Functional connectivity (FC) analysis based on rs-fMRI is often applied to quantify the statistical dependencies across different brain regions which are correlated over time (Friston, 2011). Initially, FC was assessed with the assumption that the connections remain unchanged at rest (Hutchison et al., 2013). Moreover, recent studies have found that FC is extremely useful in gender classification (Zhang et al., 2018) and individual prediction of cognition traits such as fluid intelligence (Finn et al., 2015).

However, increasing evidence has suggested that the statistical properties of FC change over different time scales across task states (Elton and Gao, 2015; Fatima et al., 2016) and during periods of unconstrained rest (Chang and Glover, 2010; Allen et al., 2014), i.e., dynamic functional connectivity (dFC). So far, spatiotemporal patterns and dynamics of functional networks derived from rs-fMRI data have been widely studied. The evidence suggested that dynamic interactions of different functional networks were relate to specific tasks (Yuan et al., 2018a; Li et al., 2019) and brain disorders such as Alzheimer’s Disease (Huang et al., 2017), which motivated us to explore the spatiotemporal characteristics of functional networks based on dFC. Moreover, dFC (Sakoğlu et al., 2010) has been successfully applied to characterizing neuropsychiatric disorders like schizophrenia (Damaraju et al., 2014; Du et al., 2017), autism spectrum disorder (Zhu et al., 2016) and depression (Demirtaş et al., 2016). Importantly, dFC is linked to individual characteristics such as cognitive flexibility (Douw et al., 2016), emotions (Tobia et al., 2017), and age (Qin et al., 2015; Davison et al., 2016), and even the variability of dFC can be used to predict high-level cognitive performance (Liu et al., 2018).

Although these studies discriminated individual cognitive ability or demographic characteristics based on dFC, most of them employed statistical characteristics of the original dynamic correlation series as features to achieve classification or prediction. Nevertheless, these manually selected features neglected the time-varying patterns in correlation timecourse of FC through folding temporal dimension of dFC, likely causing the loss of some useful information related to individual cognition traits. Actually, the temporal evolution of connectome-scale brain network interactions has been observed to fit well the task-fMRI data (Yuan et al., 2018a), suggesting the neurophysiological significance of spatiotemporal structures in functional network dynamics. Some recent studies also demonstrated the potential of temporal sequence of windowed functional correlations in predicting individual cognitive traits and demographics. For example, the time series of dFC has long-range sequential correlations that vary across the human adult lifespan (Battaglia et al., 2020) and specific temporal structures of several FC microstates have been reported to be subject-specific and heritable, and significantly linked to individual cognitive traits (Vidaurre et al., 2017). Moreover, network switching in dFC is related to task performance and sleep (Pedersen et al., 2018), attention (Madhyastha et al., 2015), schizophrenia (Damaraju et al., 2014), and depression (Zheng et al., 2017). In particular, a growing body of researches links observed patterns of non-stationary switching between FC states with aspects of the underlying neural dynamics (Hansen et al., 2015; Thompson, 2018), indicating short-term alteration in FC time series along with shifting in cognitive states. However, given that the mechanism underlying spontaneous fluctuation in resting-state dFC has not been fully understood, explicitly modeling the sequence of time-resolved FC is still a challenging task.

Currently, deep learning has undergone unprecedented development in neuroscience. The advantage of an end-to-end model like deep learning is automatically extracting abstract spatial-temporal structures from neuroimaging data (Huang et al., 2017; Li et al., 2019), which has been used for discriminating complex mental disorders (Yan et al., 2017; Zeng et al., 2018) and gender classification (Yuan et al., 2018b). In particular, RNN models, such as long short-term memory (LSTM), can effectively extract complex and non-linear time-varying patterns underlying signals’ fluctuations in a data-driven way due to its advantages of “deep in time,” and have been successfully used in identifying autism (Dvornek et al., 2017) and discriminating schizophrenia (Yan et al., 2019) from healthy controls.

In this work, we ask to what extent the dFC can be used to predict individual cognition traits including gender classification and individual prediction of fluid and crystallized intelligence, using a deep network model on the dFC time course. We proposed the use of a convolutional neural network (CNN) and LSTM framework to identify the spatial and temporal structures underlying the spontaneous fluctuation of dFC. We assumed this framework directly working on the temporal series of dFC, could avoid information reduction and make full use of high-level spatiotemporal information of dFC. This model consists of two parts. The first part includes a multi-scale 1D convolutional layer, concatenation layer, and max-pooling layer, which were designed for spatial feature extraction. The second part involves two stacked LSTM layers, which were used to detect temporal dynamics (by learning short-term sequential switching and unknown long-term/non-linear patterns) in time series of windowed correlations. Finally, the average outputs of the LSTM layer were put as the input for the final fully connected layer. We further assessed the capacity of the CNN + LSTM model on one gender classification task and two prediction tasks of individual intelligence, with comparison to support vector machine (SVM) and support vector regression (SVR) models that usually use statistical characteristics of dFC as features (Liu et al., 2018). Furthermore, we conducted deconvolutional computation to visualize the learned features corresponding to task-related connections in three tasks for demonstrating the validity of the network in learning dynamics of time-resolved FC.

## Materials and Methods

### Participants and Data Acquisition

The dataset was selected from the publicly available S1200 release of the Human Connectome Project dataset (HCP), including 1,206 subjects (age 22–35). The rs-fMRI data of all subjects was scanned in two sessions on two different days. Each session contains a right-left (RL) phase-encoding run and a left-right (LR) phase-encoding run. In addition, the HCP collected many behavioral measures, such as fluid intelligence and crystallized intelligence, which allows us to investigate the relationship between individual traits and their neuroimaging data. 1,050 subjects (gender: 569 females and 481 males) were left for this study by restricting subjects with at least one run and two intelligence measures.

Data was acquired using a 3.0 T Siemens scanner at Washington University at St. Louis. The data acquisition parameters were as follows: repetition time (TR) = 720 ms, time echo (TE) = 33.1 ms, flip angle (FA) = 52°, resolution = 2.0 mm, field of view (FOV) = 208 × 180 mm (RO × PE), matrix size = 104 × 90 (RO × PE), slices = 72, and volumes = 1,200. More details are available in the previous literature (Van Essen et al., 2013).

### Data Preprocessing

The HCP dataset was first preprocessed under the HCP minimal preprocessing pipeline, which mainly includes distortion and spatial artifact removal, motion correction, within-subject cross-modal registration, and cross-subject registration to a standard space (Glasser et al., 2013). In addition, standard preprocessing procedures for resting-state connectivity analysis were performed on the HCP using SPM8^1^. For each subject, the fMRI images were resampled to 3 × 3 × 3 mm isotropic voxels. Next, a Gaussian filter kernel of 6 mm full width at half maximum (FWHM) was used to smooth the images spatially. Then, the images were temporally bandpass filtered from 0.01 to 0.08 Hz. Finally, to further denoise rs-fMRI data, we regressed the white matter (WM) signal, head motion, cerebrospinal fluid (CSF) signal, and their first-order deviations.

### Dynamic Functional Connectivity

**FIGURE 1:**
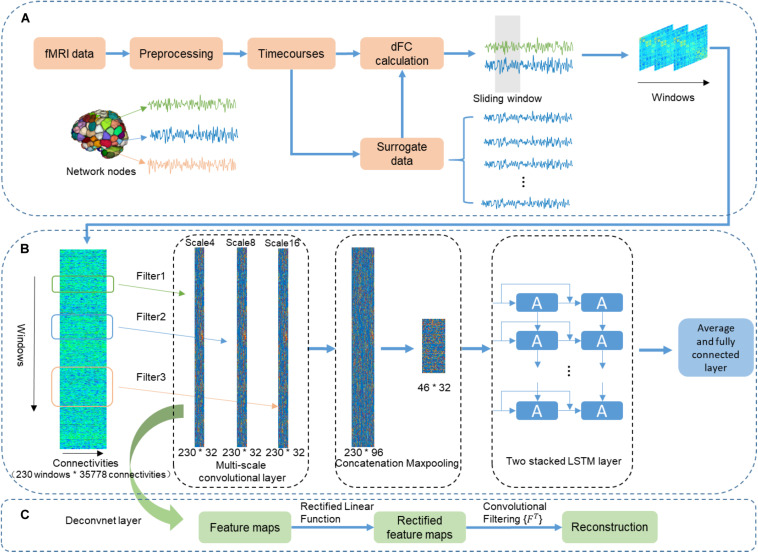
The flowchart of using the CNN + LSTM model to distinguish sex and predict intelligence based on dynamic functional connectivity (dFC). **(A)** The pipeline of calculating dFC using resting-state fMRI data and surrogate data that was generated by employing the multivariate phase randomization (MVPR) model on original BOLD signals. **(B)** Overview of multi-layer structure in the CNN + LSTM model. **(C)** The deconvolutional computation for visualizing learned spatiotemporal features in dFC from the Conv1D layers.

To obtain regions of interest (ROI) based bold signals, we averaged preprocessed rs-fMRI time courses of voxels within each gray matter region according to a 268-node functional atlas (Shen et al., 2013). Then, a sliding time window approach (Calhoun et al., 2014; Qin et al., 2015) was used to divide the ROI-based brain signals into temporal segments with a window size of 39.6 s (55 TRs), and the dFC of each region pair was calculated using Pearson correlation coefficients. As a result, a series of 268 × 268 correlation matrices were generated (Figure 1A). Additionally, to normalize the coefficient values of correlation matrices, we applied Fisher’s *z*-transformation to each correlation matrix. Next, the upper triangular part of each correlation matrix was reshaped into a vector for the following analysis. Ultimately, for each run of each subject, we obtained a sliding-window correlation matrix of 230 (windows) × 35,778 (connections).

### dFC-Based Prediction Setup

Gender and behavioral data of intelligence on high-level cognition from HCP protocol were selected, including fluid intelligence (Penn Progressive Matrices, HCP: PMAT24_A_CR) and crystallized intelligence which was measured using NIH toolbox composite scores (Crystallized Cognition Composite), combining Picture Vocabulary Test and Oral Reading Recognition Test into one score (CompGcScore) (Akshoomoff et al., 2013). Next, the gender and intelligence measures of subjects were predicted using the CNN + LSTM model based on dFC matrices.

### CNN + LSTM Model

As shown in Figure 1B, the used CNN + LSTM network is composed of three 1D convolutional layers which have three filters of different size (4, 8, and 16 time windows), one concatenation layer combining features from three convolutional layers, one max-pooling layer which was designed to down-sample, two LSTM network layers, and a fully connected layer (Yan et al., 2019). The model was built for both the gender classification task and cognitive performance prediction tasks. Specifically, the obtained dFC matrices were fed into the CNN + LSTM model as inputs for parameter optimization. Then, the optimized model was saved for testing and comparison.

#### Multi-Scale Convolutional Layer

Although LSTM is powerful for handling temporal correlation, the major drawback of LSTM in handling spatiotemporal data was the redundancy of high-dimensional data. In addition, in the previous CNN-RNN architecture, the capability of each Conv1D layer determined by a single fixed filter size is extracting local information at only one time scale. Moreover, to adapt specific tasks, the filter length of 1D convolutional layer should be hand-picked. Therefore, multi-scale 1D convolutional layers were proposed for feature extraction because it can not only reduce spatial dimension but also account for different scales of brain activity (Roy et al., 2019; Yan et al., 2019). The architecture of multi-scale 1D convolutional layers includes multiple filters with diverse sizes in each convolutional layer. Filter lengths of 1D convolutional layers changed exponentially rather than linearly. Our experiment demonstrated that this architecture led to better performance by using different filter lengths (4, 8, and 16 time windows) in the 1D convolutional layers. Therefore, the size of the convolutional filters of three different scales 1D convolutional layers are 35,778 × 4 × 32, 35,778 × 8 × 32, 35,778 × 16 × 32, respectively. Here, we fed the dFC matrices into the multi-scale convolutional layer. As a result, outputs are three features whose sizes are 230 (time windows) × 32 (feature dimension). Then, a concatenation layer is designed to concatenate the output features in the time dimension, resulting in a feature map with a size of 230 × 96. Furthermore, a downsampling operation is performed using the max-pooling layer along the time dimension with kernel size 5 × 1, resulting in 46 × 96 features as an input of the following LSTM layers.

We performed deconvolutional computation for each convolutional layer (Figure 1C) (Zeiler and Fergus, 2014) to obtain the distribution of those connections with relatively high weights in discrimination. The deconvolutional results of one 1D convolutional layer were obtained for 100 randomly selected subjects. The group-level statistical strength of each functional connection was generated by computing the absolute value of its average strength across 100 randomly selected subjects (Zhang et al., 2018).

#### Two-Layer Stacked LSTM Layer

Long short-term memory is one kind of RNN models, which is different from CNN because of its consideration of the temporal information. LSTM consists of an input gate, an output gate, a forget gate and a cell. The advantage of LSTM is that it has sufficient ability to solve long-term dependencies because of the interactive operation among these three gates, in contrast to general RNNs. In addition, LSTM is designed to combat vanishing/exploding gradients by using a gating mechanism. The LSTM model can be presented in the following form:

(1) $$F\,o\,r\,g\,e\,t\,g\,a\,t\,e:f_{t}=\text{Sigmoid}\,\left(W_{f}\,x_{t}+U_{f}\,h_{t-1}+b_{f}\right)$$

(2) $$I\,n\,p\,u\,t\,g\,a\,t\,e:i_{t}=\text{Sigmoid}\,\left(W_{i}\,x_{t}+U_{i}\,h_{t-1}+b_{i}\right)$$

(3) $$O\,u\,t\,p\,u\,t\,g\,a\,t\,e:o_{t}=\text{Sigmoid}\,\left(W_{o}\,x_{t}+U_{o}\,h_{t-1}+b_{o}\right)$$

(4) $$E\,s\,t\,i\,m\,a\,t\,e\,d\,c\,u\,r\,r\,e\,n\,t\,c\,e\,l\,l\,s\,t\,a\,t\,e:{\tilde{C}}_{t}=T\,a\,n\,h\,\left(W_{c}\,x_{t}+U_{c}\,h_{t-1}+b_{c}\right)$$

(5) $$C\,e\,l\,l\,s\,t\,a\,t\,e:C_{t}=i_{t}⊙{\tilde{C}}_{t}+f_{t}⊙C_{t-1}$$

(6) $$H\,i\,d\,d\,e\,n\,s\,t\,a\,t\,e:h_{t}=o_{t}⊙\tanh\left(C_{t}\right)$$

Where *W*_*f*_, *W*_*i*_, *W*_*o*_, and *W*_*c*_ are input weights; *U*_*f*_, *U*_*i*_, *U*_*o*_, and *U*_*c*_ are recurrent weights; *b*_*f*_, *b*_*i*_, *b*_*o*_, and *b*_*c*_ are bias weights, and ⊙ is the Hadamard product.

Here, we choose two densely connected LSTM layers because it may better capture latent dynamic information than one LSTM layer. It is worth noting that densely connected LSTM layers may mitigate the vanishing or exploding gradients problem (Roy et al., 2019; Yan et al., 2019). The size of the hidden state was set as 32. Also, we averaged all of the LSTM outputs to combine all fMRI steps (Dvornek et al., 2017). In this way, better classification performance could be obtained through leveraging all brain activities during scanning. Then the learned features were passed to the fully connected layer. The fully connected layer can be expressed as:

(7) $$h^{l}=b^{l}+h^{l-1}\times w^{l}$$

Where *w^l^* and *b^l^* are input weight and bias weight, respectively. When the model is trained for intelligence prediction, the output of the fully connected layer is the predicted intelligence scores. However, for gender classification, another operation of Softmax was added as the last operation of this architecture.

### Training, Validation, and Testing

A 10-fold cross-validation procedure was used for evaluating prediction performance. The HCP data was randomly split into training, validation, and testing sets. More specifically, we divided the 1,050 subjects into ten folds. Note that multiple runs belonging to the same subject were not split across folds (He et al., 2020). For every test fold, the remaining nine folds were used for training and validation. Furthermore, it has been found that head motion was correlated with behavioral measures such as fluid intelligence (Siegel et al., 2017). Therefore, we regressed sex, age, and motion (frame-wise displacement FD) from the intelligence. In each test fold, we estimated the regression coefficients from the training set and applied them into the test fold (Kong et al., 2019; He et al., 2020).

The CNN + LSTM model was coded based on the platform of Pytorch (Paszke et al., 2017), and optimized with Adam optimizer to minimize the loss (Yan et al., 2019). The value of batch size was set as 64. The initial learning rate was 0.0001. We decreased the learning rate with weight decay rate of 10^−1^ after each epoch. To avoid overfitting and achieve higher generalization performance, we used dropout (dropout rate = 0.5) for regulating the model parameters and early stopping to stop training according to the prediction condition of the validation data. Briefly, when training loss continued to decrease but validation loss increased, this means that the training was already overfitting, and we should stop training.

### Evaluation of Model Ability to Capture Dynamics of FC With Surrogate Data

It was unclear whether the CNN + LSTM model concentrated on the dFC containing the sequential temporal dynamics, or just captured the static statistics of dFC. To validate the validity of the model in capture dynamic interaction information, we used initial BOLD signals to generate surrogate data as null hypothesis. The aim of surrogate data is to generate the same time probability distribution, while preserving all statistical properties of the observed data like stationary cross correlation, but to destroy the dynamics in FC time courses (Schreiber and Schmitz, 2000; Pereda et al., 2005; Savva et al., 2019).

In this study, a multivariate phase randomization (MVPR) model (Prichard and Theiler, 1994) was applied by randomly shuffling the Fourier phases of the original BOLD signals such that their static FC structure could be preserved (Hindriks et al., 2016; Liégeois et al., 2017; Savva et al., 2019).

(8) $${\hat{X}}_{k}=X_{k}\,e^{i\,\varphi },k=1,2,\cdots ,n$$

Where *X* = [*X*_1_,*X*_2_,⋯,*X*_*n*_] is the discrete Fourier transformations of original time series. *n* = 268 refers to the number of brain regions. φ = [φ_1_,φ_2_,⋯,φ_*T*_] is a uniformly distributed random phase in the range of [0,2π].

Subsequently, the inverse Fourier transform is applied to ${\hat{X}}_{1},{\hat{X}}_{2},\cdots ,{\hat{X}}_{n}$ to generate one randomized copy $\hat{x}$. We repeated the procedure and then generated 100 surrogate copies for each subject. In addition, dFC matrices were calculated in the same way, excluding that surrogate copies instead of BOLD signals were used. For each surrogate copy of all subjects, the resulted dFC matrices were used as input of the model for gender classification with 10-fold cross-validation strategies. As a result, 100 surrogate copies were performed to estimate a distribution of accuracies under the null hypothesis of dFC.

Furthermore, a statistical method was proposed to assess the existence of dFC between a pair of ROIs (Savva et al., 2019). Then, this statistical framework was applied to test if the CNN + LSTM model could concentrate on the ROI pairs with statistically significant dFC. For details, 250 surrogate copies of one randomly selected participant were generated with the aforementioned method to formulate the null hypothesis. This null hypothesis can be rejected when any given FC time-series exhibits dFC.

### Evaluation of Model Performance in Gender Classification and Intelligence Prediction Tasks

As commonly seen in the recent intelligence prediction studies based on rs-fMRI data (Finn et al., 2015; Liu et al., 2018; He et al., 2020), the Pearson’s correlation between predicted and observed intelligence scores of all subjects across all folds was used for assessing the model performance of intelligence prediction tasks. In this study, we reported the Pearson’s correlation and mean absolute error (MAE) to evaluate our model prediction performance. In the case of gender classification, the model was evaluated with the classification accuracy. Additionally, the area under ROC curve (AUC) which is a very widely used measure of performance for classification was also reported. Furthermore, it has been revealed that chronnectome (Calhoun et al., 2014) could be used to identify individuals and predict individual higher cognitive performance (Liu et al., 2018). For comparing with the CNN + LSTM model, we used linear SVM and linear epsilon SVR models (LIBSVM toolbox in Matlab^2^) based on dynamic characteristic of dFC (dFC-Str, which refers to the overall strength of dFC) (Liu et al., 2018), to achieve gender classification and intelligence prediction with the same 10-fold cross-validation strategies. The hyperparameter of linear SVM and linear epsilon-SVR includes the regularization parameter *C*, which was optimized for achieving its best performance.

### Control Analysis

We further examined the effects of parcellation schemes and sliding window sizes on model performance. The selection of window size was controversial in previous studies (Kiviniemi et al., 2011; Jones et al., 2012; Hutchison et al., 2013; Allen et al., 2014; Qin et al., 2015). A smaller window size can better detect the potential low-frequency fluctuations in FC (Sakoğlu et al., 2010; Hutchison et al., 2012). It has been suggested that the window sizes from around 30–60 s can achieve the best classification and prediction performances (Shirer et al., 2012; Qin et al., 2015). In this work, three additional window sizes (60, 80, and 100 s) were used to investigate the potential impact of window width on gender classification and intelligence prediction performance. Additionally, to evaluate the effects caused by different brain parcellations, two additional parcellations were used for generating dFC matrices, including Power-264 consisting of 264 ROIs (Power et al., 2011), and functional brain atlas of 160 ROIs (Dosenbach et al., 2010). Then, we re-performed the gender classification and intelligence prediction analyses using these dFC matrices based on these parcellations.

## Results

### Performance of Gender Classification and Intelligence Prediction Tasks

#### Gender Classification Results

**FIGURE 2:**
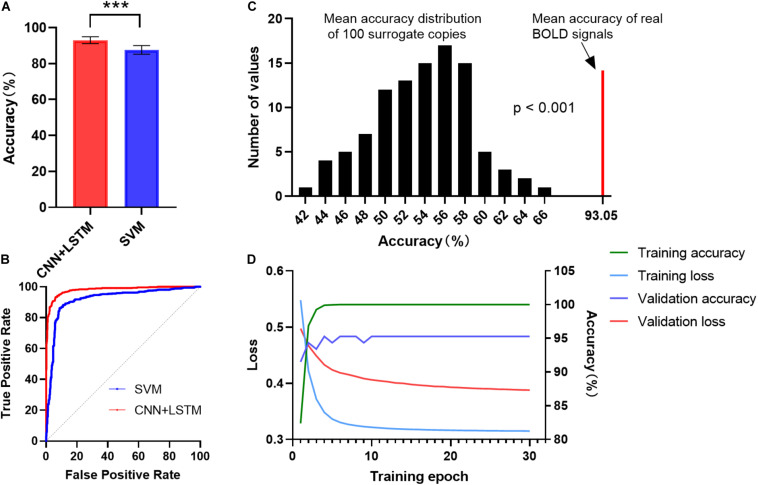
ROC and accuracy for the gender classification task. **(A)** ROC curve across 10 testing folds for the CNN + LSTM and the SVM model. **(B)** Gender classification accuracy averaged across 10 testing folds. Bars refer to mean accuracy of all testing folds. Error bars indicate the standard error. Obviously, the CNN + LSTM model is statistically better than the SVM model (***p < 0.001). **(C)** Accuracies of gender classification using the CNN + LSTM model on the real BOLD signals and their surrogate copies. A total of 100 surrogate data were generated by using MVPR to estimate the null distribution of classification accuracies (see section “Materials and Methods” for detail). With the mean classification accuracies as the statistic, results reveal that the classifier learned the connection dynamics with a probability of being wrong of <0.001. **(D)** The learning curves while training the CNN + LSTM model.

Figure 2A shows the gender classification performance of the CNN + LSTM and SVM model. The accuracy of 93.05 ± 1.91% was obtained by using the CNN + LSTM model, which is significantly higher than that obtained using SVM (*p* < 0.001, two-sample *t*-tests). Moreover, their ROC curves are shown in Figure 2B. The CNN + LSTM model achieved an AUC of 0.9805, while the SVM achieved an AUC of 0.9195. Note that the CNN + LSTM model achieved better performance than SVM by integrating the advantages of CNN and LSTM.

We conducted 100 times of repeated tests using 100 surrogate copies to testify whether the trained deep network discriminates genders based on dynamics in FC rather than other features such as static functional correlation across ROIs. The results are shown in Figure 2C. As expected, empirical distributions of the accuracies scattered around 57%, suggesting the classifier performance was just better than random guessing for the surrogate copies. All the accuracies of surrogate copies fell behind that of real BOLD signals, demonstrating that the statistical significance of gender classification based on temporal dynamics in FC was high (*p* < 0.001), so that the null hypothesis that the deep network failed in capturing spatiotemporal features of dFC could be rejected. The learning curves while training the CNN+LSTM model was shown in Figure 2D.

#### Intelligence Prediction

**FIGURE 3:**
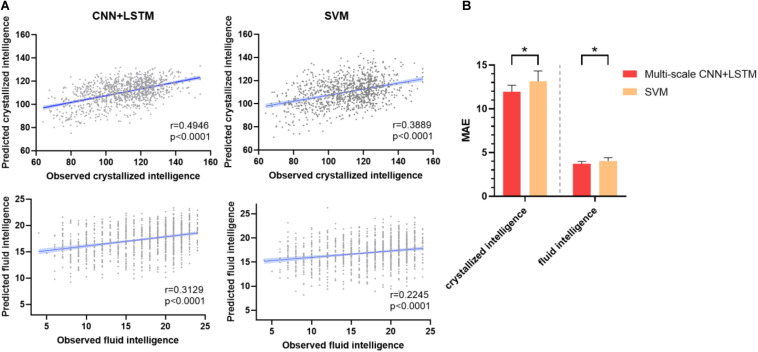
Prediction performance of fluid intelligence and crystallized intelligence. **(A)** The correlations between predicted and observed intelligence scores for the CNN + LSTM and support vector machine (SVM) models. Note that the CNN + LSTM model exhibits the highest correlation scores for both tasks. Each subject is represented by one dot, and 95% confidence interval for the best-fit line is represented by the gray area which is used to assess the predictive power of the model. **(B)** Comparison between mean MAE across 10 testing folds for the CNN + LSTM and the SVM model. Lower is better. Bars refer to the mean accuracy of all testing folds, and error bars refer to their standard error. Note that the CNN + LSTM model is statistically better than the SVM model (*p < 0.05).

Figure 3A depicts the prediction accuracy of fluid and crystallized intelligence (Pearson’s correlation coefficient) in a 10-fold cross-validation test. The CNN + LSTM model achieved higher prediction accuracy in both tasks than the SVR model, with Pearson’s correlation *r* = 0.3129 for fluid intelligence and *r* = 0.4946 for crystallized intelligence, respectively, in contrast to SVR’s prediction accuracy of *r* = 0.2245 for fluid intelligence and *r* = 0.3889 for crystallized intelligence. The conclusions of MAE are similar, as illustrated in Figure 3B. The MAE of 11.9561 ± 0.7412 (for crystallized intelligence) and 3.7287 ± 0.2673 (for fluid intelligence) in the CNN + LSTM model are significantly lower than those obtained using SVR (*p* < 0.05, two-sample *t*-tests).

### Estimating the Most Discriminative Connection Features

**FIGURE 4:**
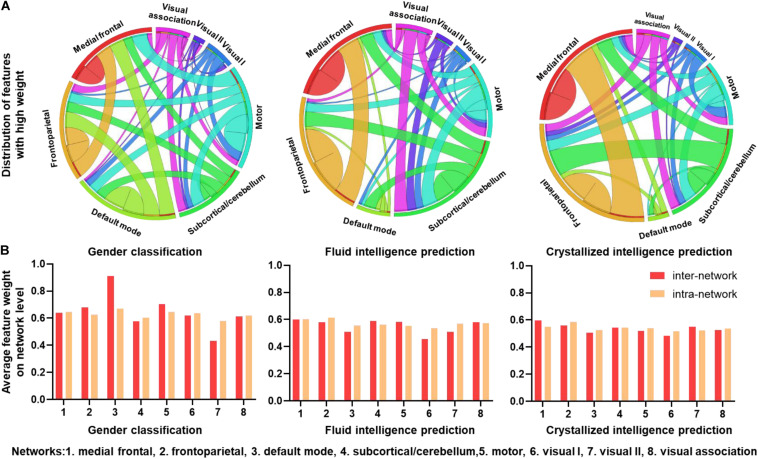
Connectivity patterns with high task-related weights. **(A)** Distributions of functional connections with feature weights that are larger than the threshold for three prediction tasks. Different brain functional networks are represented by segments of different colors, and the length of the segment refers to the total number of connections. Ribbons refer to functional connections, and the width of ribbon refers to the number of intra-network and inter-network connections. **(B)** Average feature weights of intra-network and inter-network connections for three tasks.

To explore the ability of the CNN + LSTM model in extracting features related to the three discriminating tasks, we used deconvolution computations to show the important functional connection features characterized with high weights. Figure 4A shows the distributions of FC with feature weights above the threshold, which was set for an important contribution at the 75th percentile of all feature weights. Important FC features are widespread across the brain for all three discriminating tasks. However, as for gender classification, a large number of FC features above the threshold are distributed in inter-network and intra-network of frontoparietal (networks 1 and 2), default mode (network 3), and motor (network 5), especially for the default mode and motor networks. While FC features with high weights are mostly in the inter-network and intra-network of frontoparietal for fluid and crystallized intelligence tasks.

To explore which networks had the most predictive power, average intra-network and inter-network feature weights of eight networks were calculated without thresholding. As illustrated in Figure 4B, the default mode network (DMN) has the highest inter-network and intra-network feature weights of gender classification, especially for the inter-network. The motor and frontoparietal networks follow the default mode. The other networks have slightly low weights of intra-network and inter-network features. Additionally, patterns from fluid intelligence and crystallized intelligence predictions are very similar, i.e., the frontoparietal network has slightly higher weights than the others. However, the visual I network (network 6) has low predictive power for predicting intelligence. Intra-network and inter-network feature weights are comparable in all the networks.

### Identifying Significant Dynamic Connections With Surrogate Data

**FIGURE 5:**
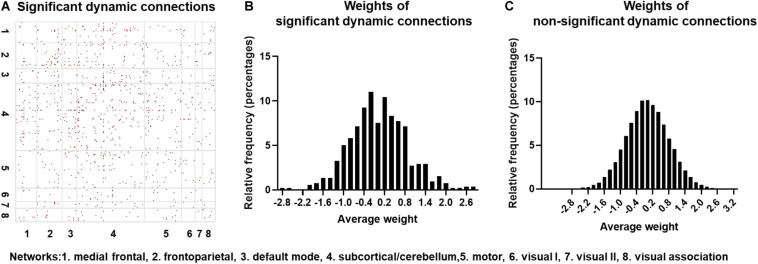
Statistically significant dynamic connections for one randomly selected subject. **(A)** Distribution of significant dynamic connections (red points) in eight networks. **(B)** Distribution histogram of deconvolutional weights for significant dynamic connections. **(C)** Distribution histogram of deconvolutional weights for non- significant dynamic connections.

The null hypothesis for dFC using the surrogate data was tested on one randomly selected subject, with the results indicated in Figure 5A. The red points represent those connections exhibiting statistically significant dFC. Note that significant connections were widely distributed across the brain. To further identify if the CNN + LSTM model could capture those regions with statistically significant dynamics, we computed the distributions of averaged deconvolutional weights for all significantly dynamic and non-significantly dynamic connections. As shown in Figures 5B,C, both the weight distributions of ROI pairs follow the approximately normal distribution. Moreover, the features with high weights cover nearly half of those connections with significant dFC. In other words, the important regions extracted by using the CNN + LSTM model carry sufficient dynamic connectivity information.

## Discussion

In this work, we used a CNN + LSTM model combining the advantages of CNN and LSTM to learn spatiotemporal information in rest-state dFC. This was the first attempt to capture the dynamic interaction information of dFC series using deep learning, which avoids information reduction and takes advantage of time-varying spatiotemporal information. More importantly, we showed that the CNN + LSTM model not only successfully achieved a high accuracy of gender classification but could significantly predict individual intelligence, including fluid and crystallized intelligence across a large-scale dataset totaling 1,050 participants. Moreover, the results of deconvolutional computation provided interpretation for extracted features. Our results suggest that the CNN + LSTM model can simultaneously learn temporal and spatial information of dFC series instead of dFC’s statistical characteristics, which could significantly improve the prediction power of individual cognition traits.

### Gender Classification and Intelligence Prediction Performance

Table 1 summarizes the rs-fMRI based model performance of gender classification and intelligence prediction from some recent studies. Zhang et al. (2018) showed that with three different methods combining FC features of multiple runs from the HCP (820 subjects), the optimal classification accuracy and AUC were 85% and 0.93, respectively. Ktena et al. (2018) achieved a gender classification accuracy of 80% with static functional connectivity (sFC) from UK Biobank (2,500 subjects). In addition, He et al. (2020) reported a sex prediction accuracy of 91.6% with four different models in the UK Biobank (8,868 subjects). Our model based on dFC time series could achieve a higher accuracy of gender classification than those using the static FC feature.

**TABLE 1:** Model performance of rs-fMRI based gender classification and intelligence prediction tasks in some recent studies.

**Author**	**Dataset (subs)**	**Methods**	**Types of data**	**Tasks**	**Accuracy**
Chao Zhang	HCP (820)	PLSR	sFC	Gender	87% (10-fold)
Sofia Ira Ktena	UK Biobank (2,500)	GCN	sFC	Gender	80% (5-fold)
Tong He	UK Biobank (8,868)	TML	sFC	Gender	91.6% (9-fold)
Casanova R	FCP (148)	Lasso regression	sFC	Gender	62%
Stephen M. Smith	HCP (131)	Linear discriminant analysis	sFC	Gender	87% (LOOCV)
Susanne Weis	HCP (434)	SVM	sFC	Gender	75.1% (10-fold)
Tong He	HCP (953)	FNN	sFC	Fluid intelligence	0.297 (20-fold)
Julien Dubois	HCP(884)	Linear models	sFC	Fluid intelligence	0.27 (leave-one family out)
Rajan Kashyap	HCP (803)	COBE	sFC	Fluid intelligence	0.25 (20-fold)
Ru Kong	HCP (881)	MS-HBM	sFC	Fluid intelligence	0.22 (20-fold)
Abigail S. Greene	HCP (515)	CPM	sFC	Fluid intelligence	0.196 (LOOCV)
Jin Liu	HCP (105)	SVR	dFC	Fluid intelligence	0.418 (LOOCV)
Emily S. Finn	HCP (142)	Linear regression	sFC	Fluid intelligence	0.5 (LOOCV)

*All results are the best performance mentioned in the literature. CNN, convolutional neural network; GCN, graph convolutional network; PLSR, partial least squares regression; COBE, common and orthogonal basis extraction; MS-HBM, multi-session hierarchical Bayesian model; CPM, connectome-based predictive modeling; LOOCV, leave-one-subject-out cross-validation.*

In the case of behavioral measures such as fluid and crystallized intelligence, combining Picture Vocabulary Test (vocabulary) and Oral Reading Recognition Test (reading) into one score, previous studies (Finn et al., 2015; Liu et al., 2018) reporting high fluid intelligence prediction accuracy were both using a leave-one-subject-out cross-validation for small samples from the HCP dataset (126 subjects and 105 subjects). Recent studies reported lower accuracies when the number of samples increased. For example, He et al. (2020) reported prediction accuracies (Pearson’s correlation) ranging from 0.257 to 0.297 for fluid intelligence, about from 0.25 to 0.4 for reading and approximately from 0.2 to 0.4 for vocabulary, with four different models in the HCP dataset (953 subjects). Dubois et al. (2018) successfully predicted fluid intelligence (*r* = 0.27) using linear models in the HCP dataset (884 subjects). Kashyap et al. (2019) reported prediction accuracies of 58 behavioral measures, including fluid intelligence (*r* = 0.25), reading (about *r* = 0.37) and vocabulary (about *r* = 0.39) in the HCP dataset (803 subjects).

It is worthy to note that all these methods are based on the sFC, assuming that the underlying connections remain unchanged over time. Here, we applied a CNN + LSTM model to learn the spatiotemporal information of the original dFC series. Importantly, combining the multiple 1D convolutional layers of different filters can learn the spatial associations of dFC at different time scales. And the stacked LSTM layers can capture latent temporal dynamics. As mentioned earlier, we achieved a gender classification accuracy of 93.05% and prediction accuracies (Pearson’s correlation) of 0.3129 and 0.4946 for fluid intelligence and crystallized intelligence, respectively. Overall, this indicates that our model prediction performance is among the best in the literature based on the same size dataset of rs-fMRI. Further, we employed an ablation control analysis to highlight necessity of the use of the LSTM module, i.e., we only retained the multi-scale 1D convolutional layer, one concatenation layer and one fully connected layer to reperform the intelligence prediction tasks. Other parameters are consistent with the CNN + LSTM model. As shown in Supplementary Figure S1, the multi-scale CNN model only achieved a significantly low prediction accuracy of crystallized intelligence (Pearson’s correlation *r* = 0.1866) and fluid intelligence (*r* = 0.1277), suggesting that the LSTM greatly contributed to the prediction of individual intelligence by capturing the temporal information of dFC time series.

### Effectiveness Analysis Using Surrogate Data

So far, many dFC studies have employed MVPR model to generate surrogate data for detecting significance of dynamics in resting-state FC (Hindriks et al., 2016; Savva et al., 2019) by preserving statistical properties of the initial data such as stationary cross-correlation, i.e., static FC structure. Given that the dynamic information is removed in the surrogate data, the null hypothesis is that the model can achieve high classification or prediction accuracy in the absence of the dFC. However, our results demonstrate that the classification performances of surrogate copies are just better than random guessing, so that the null hypothesis should be rejected (*p* < 0.0001). Moreover, the results from the feature analysis in the model also provide further evidence supporting this deduction, i.e., the features with high weights from the 1D convolution layer covered nearly half of those connections with significant dFC, especially those connections with the highest weights. These outcomes from the surrogate data suggest that the CNN + LSTM model can sufficiently learn temporal dynamics rather than only static structure in the FC.

### Important Networks of Gender and Intelligence Discrimination

As shown in Figure 4, the FC features within the DMN‘, frontoparietal and motor networks had a great contribution to gender classification, especially, the DMN has the highest inter-network and intra-network feature weights, which is generally consistent with previous structural and functional MRI studies (Zhang et al., 2018; Luo et al., 2019). However, the subcortical/cerebellum and visual networks are the majority of the least discriminative functional networks.

Previous researches have shown that the DMN is related to many different functions like social understanding (Li et al., 2014), social cognitive abilities (Schilbach et al., 2008; Mars et al., 2012), and episodic memory (Sestieri et al., 2011). While many studies reported sex differences in behavioral measures. For example, women perform better than men on memory tasks (Hedges and Nowell, 1995) as well as measures of social cognition (Gur et al., 2010, 2012; Moore et al., 2015). These results revealed the underlying associations between the DMN and gender.

Besides the DMN, there were also other networks with high contributions to the gender classification. For instance, features with high weights for gender classification are prominent in the frontoparietal and motor networks. Also, the network-level average feature weight was illustrated in Figure 5B, indicating that the intra-network and inter-network weights of the motor and frontoparietal regions are slightly higher than other networks except for the DMN. Recent studies have also reported that most FC features within frontalparietal and sensorimotor networks are associated with gender differences (Zhang et al., 2016, 2018). Additionally, the reliable gender difference in FC has been reported for the sensorimotor network (Weis et al., 2019).

In the case of fluid intelligence and crystallized intelligence, we found stronger FC features are mostly associated with the frontoparietal network. This suggests that individual variability in intelligence is related to higher order systems that reflect individual cognitive ability than those of the primary systems. Similarly, Liu et al. (2018) found that dFC features of default mode, frontoparietal, and dorsal attention networks contributed predominantly to fluid intelligence and executive function. The frontoparietal network was also reported to be the most predictive of fluid intelligence (Finn et al., 2015). Moreover, many previous studies also reported that intelligence is related to the structural and functional properties of these networks, which are consistent with ours (Kanai and Rees, 2011; Cole et al., 2012). On the other hand, for the intelligence prediction tasks, all the networks have comparable average weights, suggesting strong evidence that more than one network accounts for the intelligence, consistent with the previous report that intelligence is related to functional coupling and structural connectivity across widespread brain regions (Choi et al., 2008).

### Effect of Parcellation Scheme and Sliding Window Width

**FIGURE 6:**
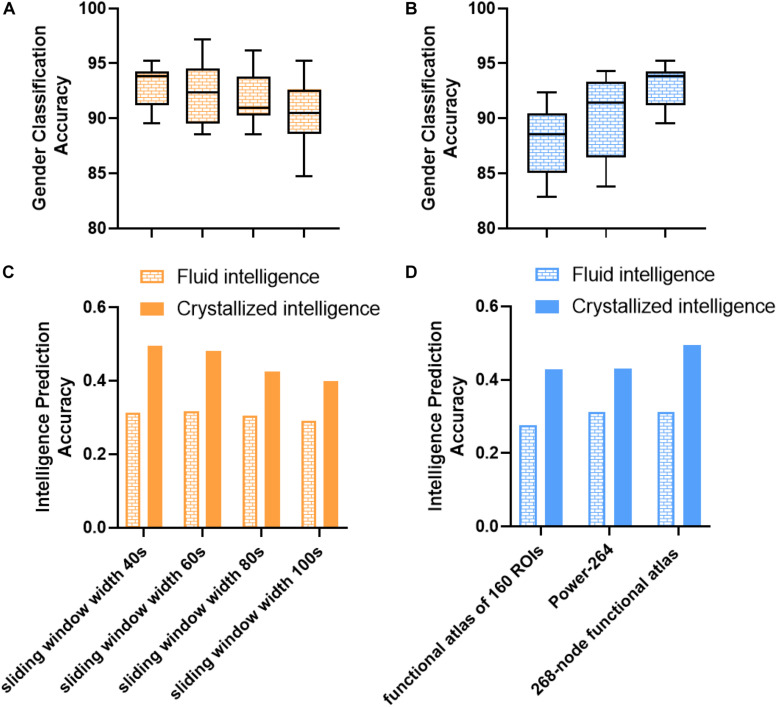
Impact of parcellation scheme and sliding window width on gender classification and intelligence prediction performance in control analysis. **(A)** Impact of sliding window width on gender classification. **(B)** Impact of parcellation scheme on gender classification. Box refers to median with 25 and 75th percentiles, and whiskers represent minimal to maximal values. **(C)** Impact of sliding window width on intelligence prediction. **(D)** Impact of parcellation scheme on intelligence prediction. The accuracy of intelligence prediction is evaluated with the Pearson’s correlation between predicted and observed intelligence scores of all subjects across all folds.

Several confounding factors, such as sliding window width and parcellation scheme, might influence prediction performance. As shown in Figure 6, we investigated the impact of the parcellation scheme and sliding window width and found that most of the results remained robust. All the sliding window widths from 40 to 100 s could achieve a robust prediction performance, thus providing indirect evidence of dFC’s presence at different timescales (Savva et al., 2019). Intriguingly, the window width of 40 s achieved the best performance in gender classification and crystallized intelligence prediction tasks. The window width of 60 s in fluid intelligence prediction task achieved a slightly higher performance (*r* = 0.3172) than the window width of 40 s (*r* = 0.3129). These results suggested that the duration between 40 and 60 s could better capture dynamic information of fluctuations and decode the variability of individuals, in line with the previous findings (Shirer et al., 2012; Kucyi and Davis, 2014; Qin et al., 2015). However, the mean accuracies of gender classification based on the functional brain atlas of 160 ROIs and 264 ROIs were about 88 and 90.3%. The prediction accuracies of intelligence prediction tasks based on the functional brain atlas of 160 ROIs and 264 ROIs were 0.2758 and 0.3120 for fluid intelligence, 0.4283 and 0.4308 for crystallized intelligence, respectively. Reduction in gender classification and intelligence prediction accuracies compared to the 268-node functional parcellation suggests that a finer parcellation may detect more subtle individual variability and dynamics.

### Limitation and Conclusion

Some limitations should be considered in the presented approach. Firstly, We used the gender classification task to demonstrate the effectiveness of the deep learning model, considering that gender is an accurate label and many recent studies have focused on gender classification (Smith et al., 2013; Ktena et al., 2018; Zhang et al., 2018). However, compared to SVM, the improvement (about 5%) of gender classification is considerate but still limited which may be caused by the ceiling effect of gender classification. A better way of evaluating the model is to extend the model to applications of individual diagnosis for typical mental disorders such as schizophrenia and depression. Many recent studies aimed to find neuroimaging-based biomarkers for disease diagnosis using deep learning techniques. For example, In our previous study (Zeng et al., 2018), the deep autoencoder network achieved an improvement (>5.0%) in diagnosing schizophrenia across multiple imaging sites compared to other multi-site studies such as using linear classifiers (Skåtun et al., 2017). Furthermore, LSTM also shows potential capabilities in diagnosing disease. Dvornek et al. (2017) achieved a classification accuracy of 68.5% in discriminating autism spectrum disorders with LSTMs, which demonstrated a higher classification accuracy compared to previous studies (Ghiassian et al., 2016; Abraham et al., 2017). Overall, based on the findings of abnormal functional connectivities in psychiatric disorders (Damaraju et al., 2014; Yan et al., 2017; Zeng et al., 2018) and the potential abilities of deep learning in disease diagnosis, the CNN + LSTM model may have excellent prospects in assistant diagnosis of some mental disorders.

Secondly, we applied the CNN + LSTM model to explore brain network dynamics only with rs-fMRI data. While multi-modalities fusion (e.g., combining electroencephalography measures) will be helpful for generating a more accurate model due to the higher temporal resolution of electroencephalography data relative to fMRI signals. Thirdly, interpretation of the LSTM model remains unclear but a critical field of research. We will further aim to interpret deep learning models in the future.

In summary, this is the first time that original dFC series are successfully applied to discriminating individual cognitive ability or demographic characteristics such as sex, fluid, and crystallized intelligence. Furthermore, the high accuracies in these tasks indicated the effectiveness of the used model owing to the full use of the high-level spatiotemporal information of dFC. Also, the deconvolutional computation provides an interpretation of the deep learning methods. Our findings highlight that deep-chronnectome can capture the inherent dynamical interaction information of functional networks and provide the potentials for predicting individualized characterization of demographics and cognition traits.

## Data Availability Statement

Publicly available datasets were analyzed in this study. This data can be found here: https://www.humanconnectome.org.

## Author Contributions

DH and HS designed the study. JQ and LF performed the experiments. LF, JS, and HS discussed the results and contributed to the final manuscript. All authors contributed to the article and approved the submitted version.

## Conflict of Interest

The authors declare that the research was conducted in the absence of any commercial or financial relationships that could be construed as a potential conflict of interest.

## Footnotes

1 http://www.fil.ion.ucl.ac.uk/spm

2 http://www.csie.ntu.edu.tw/~cjlin/libsvm
