# Supplementary material for: A Deep Network Model on Dynamic Functional Connectivity With Applications to Gender Classification and Intelligence Prediction
Source: Front Neurosci. 2020 Aug 18;14:881. doi: 10.3389/fnins.2020.00881 (PMC7461846; doi:10.3389/fnins.2020.00881)
Supplement: Supplementary file 1 [file Image_1.pdf]

A deep network model on dynamic functional connectivity with applications to gender classification and intelligence prediction

Supporting Information (SI)

Liangwei Fan, Jianpo Su, Jian Qin, Dewen Hu, Hui Shen*

College of Intelligence Science and Technology, National University of Defense Technology, Changsha, Hunan 410073, China

*** Correspondence:**
Corresponding Author
shenhui@nudt.edu.cn

Supplementary Figures

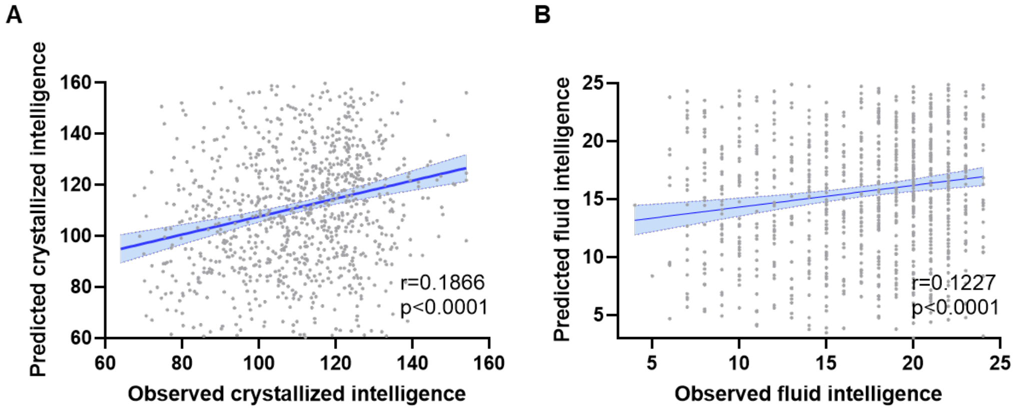

Figure S1. Prediction accuracy of fluid intelligence and crystallized intelligence tasks is greatly decreased when removing the LSTM module. (A) The correlations between predicted and observed crystallized intelligence scores for the multi-scale CNN. (B) The correlations between predicted and observed fluid intelligence scores for the multi-scale CNN. Each dot is one subject, and the gray area represents the 95% confidence interval for the best-fit line that is used to assess the predictive power of the model.
